# Profile of crosstalk between glucose and lipid metabolic disturbance and diabetic cardiomyopathy: Inflammation and oxidative stress

**DOI:** 10.3389/fendo.2022.983713

**Published:** 2022-09-15

**Authors:** Meng-Yuan Chen, Xiang-Fei Meng, Yu-Peng Han, Jia-Lin Yan, Chi Xiao, Ling-Bo Qian

**Affiliations:** School of Basic Medical Sciences & Forensic Medicine, Hangzhou Medical College, Hangzhou, China

**Keywords:** disturbance of glucose metabolism, inflammation, oxidative stress, disturbance of lipid metabolism, diabetic cardiomyopathy

## Abstract

In recent years, the risk, such as hypertension, obesity and diabetes mellitus, of cardiovascular diseases has been increasing explosively with the development of living conditions and the expansion of social psychological pressure. The disturbance of glucose and lipid metabolism contributes to both collapse of myocardial structure and cardiac dysfunction, which ultimately leads to diabetic cardiomyopathy. The pathogenesis of diabetic cardiomyopathy is multifactorial, including inflammatory cascade activation, oxidative/nitrative stress, and the following impaired Ca^2+^ handling induced by insulin resistance/hyperinsulinemia, hyperglycemia, hyperlipidemia in diabetes. Some key alterations of cellular signaling network, such as translocation of CD36 to sarcolemma, activation of NLRP3 inflammasome, up-regulation of AGE/RAGE system, and disequilibrium of micro-RNA, mediate diabetic oxidative stress/inflammation related myocardial remodeling and ventricular dysfunction in the context of glucose and lipid metabolic disturbance. Here, we summarized the detailed oxidative stress/inflammation network by which the abnormality of glucose and lipid metabolism facilitates diabetic cardiomyopathy.

## 1 Introduction

There are three major nutrients, including carbohydrates, fats and proteins, for human body’s metabolism. The monosaccharide, especially glucose, hydrolyzed from carbohydrates provide indispensable energy for cellular function and survival. Diabetes mellitus is characterized by dysregulation of insulin pathway and insulin resistance leading to the disorder of carbohydrate metabolism and hyperglycemia. Microvascular and macrovascular abnormalities, resulting in diabetic retinopathy, nephropathy, neuropathy and cardiomyopathy, are primary causes of high disability and mortality in diabetes (1, 2). Diabetic cardiomyopathy (DCM) is confirmed to be inseparably related to the abnormal myocardial energy metabolism (3). With the development of diabetes, the inhibition of glucose utilization is accompanied with the increase of fatty acid β-oxidation to satisfy the cellular energy requirement (4). Lots of cellular signaling transduction pathways associating with inflammatory response and oxidative stress are abnormally regulated in the diabetic heart and cardiomyocyte because of the disturbance of glucose and lipid metabolism, which is involved in the development of cardiac hypertrophy, fibrosis and heart failure (5, 6). Here, we summarized the crosstalk between the abnormality of glucose and lipid metabolism and DCM focusing on oxidative stress and inflammation.

## 2 What is diabetic cardiomyopathy

It has been estimated in IDF Diabetes Atlas (the 10^th^ edition) that the global number of diabetes in the age of 20-79 was 537 million in 2021 (an increase of 16% compared with that in 2019), which has been projected to reach 643 million by 2030, and more than 6.7 million adults have died (about 12.2% of global death) due to diabetes and diabetic complications in 2021 (http://diabetesatlas.org/atlas/tenth-edition/). Such a high prevalence of diabetes and a hug number of diabetes-related death confirm that diabetes is one of the fastest growing global health emergencies in the 21^st^ century. Despite the different pathophysiology of type 1 diabetes mellitus (T1DM) and T2DM, chronic systemic hyperglycemia and the disorder of glucose and lipid metabolism ultimately result in severe complications, such as blindness, kidney failure, cardiomyopathy, stroke, and lower limb amputation (2). The cardiovascular system is one of the most vulnerable targets of diabetes and suffers from endothelial dysfunction, atherosclerosis, cardiomyopathy, and even heart failure, which accounts for the major cause of morbidity and mortality in diabetic patients (7). Diabetes confers about a two-fold excess risk for cardiovascular disease independently from other conventional risk factors (8) and three-fold higher cardiovascular mortality had been reported in diabetic subjects by the Framingham Heart Study (4, 9). DCM is defined as a chronic metabolic heart disease in the absence of congenital heart disease, coronary artery disease, cardiac valve disease, or hypertension and is one of the major causes of the mortality in diabetic patients (10, 11). The typical manifest of DCM includes cardiac remodeling (hypertrophy and fibrosis) and dysfunction (12). Left ventricular diastolic dysfunction is one of the earliest characteristics of the diabetic heart and systolic dysfunction, even heart failure, develop at a later stage of DCM independent of hypertension and ischemic coronary artery disease (4, 12, 13).

## 3 Metabolic disorder in the diabetic heart

**Figure 1:**
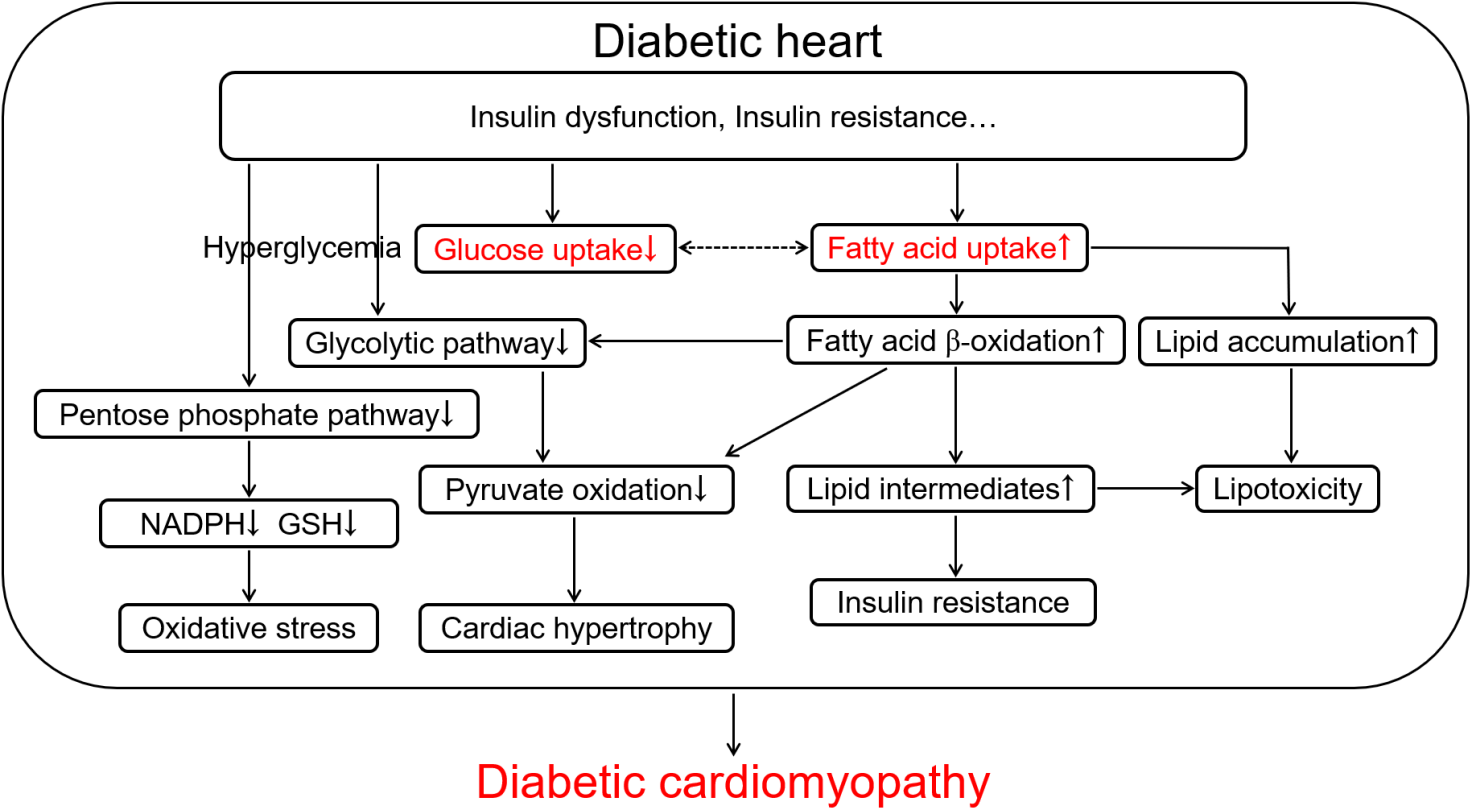
The scheme of glucose and lipid metabolism disorder in the diabetic heart contributing to diabetic cardiomyopathy. Diabetes-induced insulin dysfunction and insulin resistance cause the increase in fatty acid uptake characterized by up-regulation of fatty acid β-oxidation and lipid accumulation and the decrease in glucose uptake accompanying with hyperglycemia and down-regulation of pentose phosphate pathway and glycolytic pathway in the cardiomyocyte. Up-regulation of fatty acid β-oxidation further promotes the inhibition of glycolytic pathway and pyruvate oxidation, and increases lipid intermediates and lipotoxicity. These metabolic alterations in diabetes ultimately cause oxidative stress, further insulin resistance, and cardiac hypertrophy, which aggravates the development of diabetic cardiomyopathy. NADPH, reduced form of nicotinamide adenine dinucleotide phosphate; GSH, reduced glutathione.

Although the normal adult heart can use a variety of energy substrates, such as glucose, lactate, fatty acids, ketones, and amino acids for the continuous requirement of energy, but it primarily utilizes long-chain fatty acids (LCFA) to supply 50%-70% of energy (4, 14). Chronic alterations of myocardial substrate preference, which begins from the early stage of diabetes, can contribute to DCM. As shown in **Figure 1**, the decrease in utilization of glucose owing to insulin resistance promotes fatty acid β-oxidation to supply 90%-100% of energy associated with the translocation of cluster of differentiation 36 (CD36), a fatty acid transporter, into the sarcolemma and enhanced LCFA uptake in the cardiomyocyte, meanwhile leads to the lipotoxic cardiomyopathy in diabetes (14–16). Irreversible hyperglycemia and over-use of fatty acid β-oxidation, as forerunners in diabetes (13), break the cellular homeostasis of oxidation-reduction reaction and provoke cardiac inflammatory response, characterized by the over-production of reactive oxygen species (ROS) and pro-inflammatory factors, which contributes to the development of DCM, including ventricular remodeling, hypertrophy, myocardial stiffness, cardiac dysfunction, and heart failure (4, 13, 17). The impaired myocardial structure and function both contribute to further abnormalities of glucose and lipid metabolism in the diabetic heart, generating a positive feedback loop to exacerbate the cardiac injury in diabetes, which can not be ignored in the development of DCM (3, 18–20). Thus, rectifying metabolic disorder in the diabetic patient has been clinically accepted to attenuate cardiovascular complications.

### 3.1 Glycometabolism disorder and DCM

**Figure 2:**
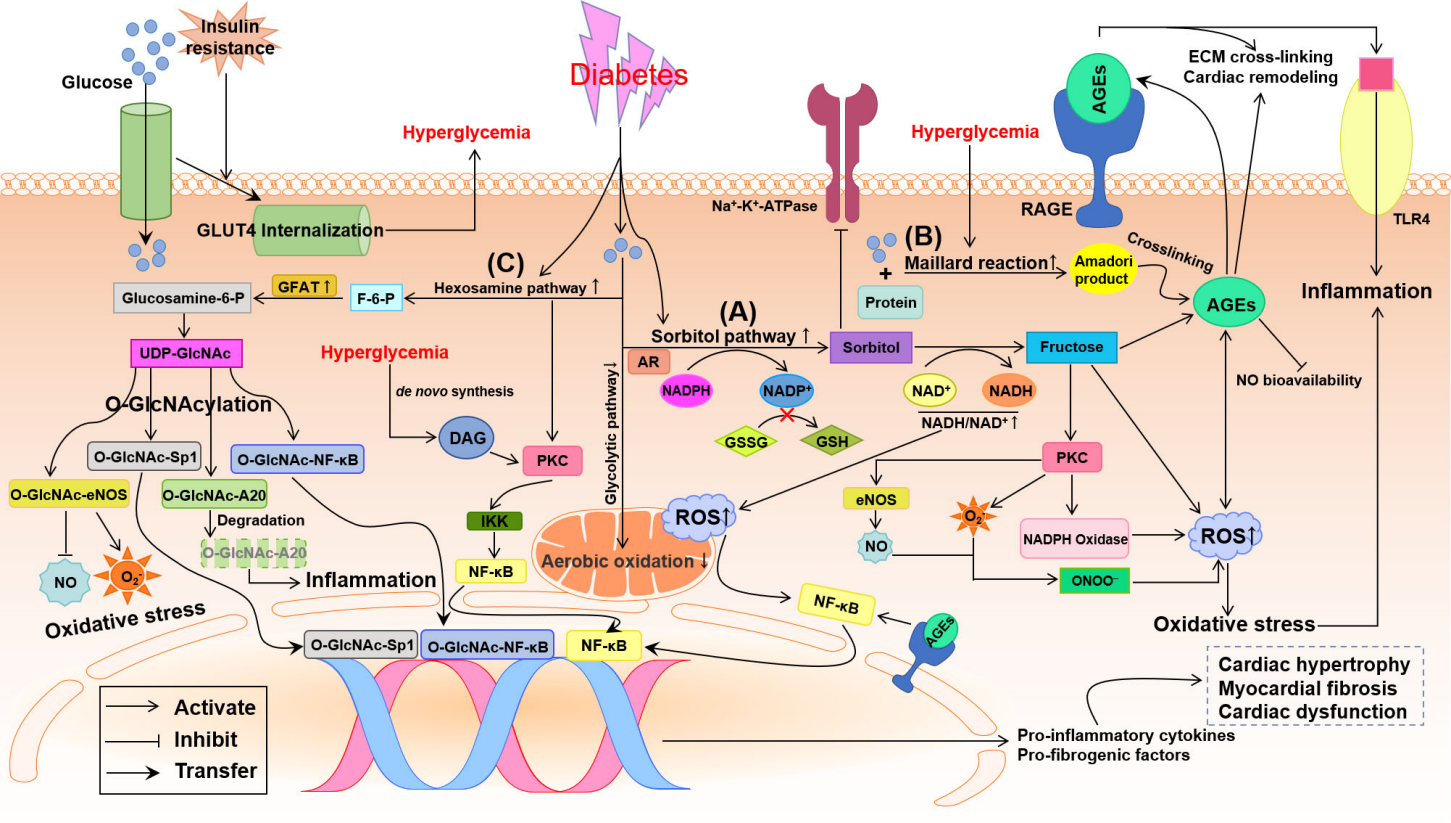
Potential pathways by which glycometabolism disorder damages the diabetic heart. Insulin resistance promotes the internalization of glucose transporter 4 (GLUT4) that means the decrease of transmembrane GLUT4 to inhibit glucose uptake in the diabetic cardiomyocyte. Oxidative stress and inflammation induced by the disturbance of glycometabolism involve the activation of three major pathways: sorbitol pathway, advanced glycation end-products (AGEs)/receptor of AGE (RAGE) and hexosamine pathway. **(A)** Up-regulation of sorbitol pathway augments oxidative stress through decreasing the reduced glutathione (GSH) and increasing reactive oxygen species (ROS), which causes nuclear factor κ-light-chain-enhancer of activated B cells (NF-κB)-induced inflammation. **(B)** Hyperglycemia boosts the formation AGEs and activates the AGE/RAGE system, which results in extracellular matrix (ECM) cross-linking, cardiac remodeling, nitric oxide (NO) inactivation and oxidative stress. **(C)** Up-regulation of hexosamine biosynthesis in diabetes leads to O-linked N-acetylglucosamine glycosylation (O-GlcNAcylation) modification of proteins, producing O-GlcNAc-A20, endothelial NO synthase (eNOS), Sp1 and NF-κB. O-GlcNAcylation of A20 accelerates its ubiquitination and proteasomal degradation resulting in inflammation. O-GlcNAcylation of Sp1 and NF-κB activate the transcriptional function to up-regulate pro-inflammatory and pro-fibrogenic factors. O-GlcNAc-eNOS decreases NO formation while increases superoxide anion (O^2-^) production. In addition, hyperglycemia may also activate protein kinase C (PKC), directly through de novo synthesis of diacylglycerol (DAG) and indirectly through increased flux of sorbitol pathway and hexosamine pathway, to promote inflammation and oxidative stress. All these glycometabolism disorders result in cardiac remodeling and dysfunction in diabetes. AR, aldose reductase; F-6-P, fructose 6-phosphate; GFAT, glutamine-fructose-6-phosphate amidotransferase; Glucosamine-6-P, glucosamine-6-phosphate; GSSH, oxidized glutathione; IKK, IκB kinase; NADH/NAD^+^, reduced/oxidized form of nicotinamide adenine dinucleotide; NADP^+^, nicotinamide adenine dinucleotide phosphate; NADPH, reduced form of NADP^+^; ONOO^-^, peroxynitrite; TLR4, toll-like receptor 4; UDP-GlcNAc, UDP-N-acetylglucosamine.

Hyperglycemia is an independent risk factor for DCM (5). The membrane glucose transporters (GLUTs) are responsible for the uptake of glucose into the cardiomyocyte. The shortage of insulin signaling or insulin resistance results in the internalization of GLUT4 and a marked down-regulation of membrane GLUT4 (**Figure 2**), which finally leads to the decreased glucose uptake in cells and hyperglycemia (21, 22) and compensatory increased LCFA uptake to break the equilibrium of myocardial energy metabolism in diabetes (5). Chronic hyperglycemia induces complicated alterations, including activation of sorbitol and hexosamine pathways, the increase of advanced glycation end-products (AGEs) and receptors for AGEs (RAGEs), and the explosion of ROS and proinflammatory factors, to result in cardiac remodeling and dysfunction in diabetes (**Figure 2**) (5, 13, 23).

#### 3.1.1 Up-regulation of sorbitol pathway

With the development of diabetes, aldose reductase (AR), a rate-limiting enzyme in sorbitol pathway, is up-regulated to convert glucose into sorbitol at the expense of reduced form of nicotinamide adenine dinucleotide phosphate (NADPH). Sorbitol is, in turn, converted into fructose by sorbitol dehydrogenase at the expense of oxidized form of nicotinamide adenine dinucleotide (NAD^+^) (**Figure 2A**) (24–26). Fructose can be further metabolized into fructose 3-phosphate and 3-deoxyglucosone, two potent nonenzymatic glycation agents, to augment the formation of AGEs, resulting in ROS production (25). Because NADPH is required for the shift of oxidized glutathione (GSSH) to reduced glutathione (GSH), an endogenous antioxidant, the consumption of NADPH by sorbitol pathway up-regulated by chronic hyperglycemia breaks the cellular antioxidant capacity (**Figure 2A**) (27). Thus the increase in intracellular sorbitol produced by sorbitol pathway has been identified as a biomarker of oxidative stress. Besides, the up-regulation of sorbitol pathway aberrant increases the activation of protein kinase C (PKC) (2). Accumulation of intracellular sorbitol causes hyperosmotic stress and the decreased activity of Na^+^-K^+^-ATPase (2, 23). An increase in cytosolic NADH/NAD^+^ triggers mitochondrial dependent ROS formation (27). All these alterations induced by the up-regulation of sorbitol pathway in diabetes augments oxidative stress and leads to diabetic cardiovascular complications (**Figure 2A**).

#### 3.1.2 Accumulation of AGEs

The AGEs formation is closely related to hyperglycemia and involves the development of diabetic cardiovascular complications (28, 29). Under long-standing hyperglycemia, reducing sugars such as glucose and fructose non-enzymatically binds with amino-acid residues in proteins, lipids and nucleic acids to form Schiff bases and Amadori products, the so-called Maillard reaction (**Figure 2B**), resulting in the accumulation of AGEs (30). The connective tissue matrix and membrane are prime targets of advanced glycation, by which high concentrations of AGEs are accumulated in cardiac tissues characterized by extracellular matrix (ECM) cross-linking (4) and myocardial stiffness (31). Formation of AGEs also produces excess ROS, which positively promotes the surges of AGEs, and reduces the bioavailability of NO leading to oxidative stress. AGEs activate RAGE to amply the inflammatory response by modulating nuclear factor κ-light-chain-enhancer of activated B cells (NF-κB) signaling and toll-like receptor 4 (TLR4) pathway (**Figure 2B**) (5, 31). These changes jointly promote myocardial fibrosis and diastolic dysfunction in diabetes. The interaction of AGE with a pattern recognition receptor termed RAGE affects numerous cell signal pathways related to inflammation and oxidative stress (31). Blocking RAGE signaling or knockdown RAGE gene has been demonstrated to alleviate cardiac hypertrophy and fibrosis and prevent the diabetic heart from systolic and diastolic dysfunction (32, 33).

#### 3.1.3 Up-regulation of hexosamine biosynthesis

Under physiological conditions, only 2%-5% of the total glucose is metabolized through the hexosamine pathway (2, 27). When exposed to persistent hyperglycemia, the hexosamine pathway and the rate-limiting enzyme, glutamine-fructose-6-phosphate amidotransferase (GFAT), are both over activated to convert glucose into excessive fructose 6-phosphate (F-6-P), glucosamine-6-phosphate and finally into UDP-N-acetylglucosamine (UDP-GlcNAc) (**Figure 2C**) (34). UDP-GlcNAc can donate N-acetylglucosamine to serine or threonine residues of target proteins within the nucleus and cytosol forming O-linked glycoproteins of proteins, which is called post-translational modification of proteins by single O-linked N-acetylglucosamine glycosylation (O-GlcNAcylation) (2, 34). The increased O-GlcNAcylation modification of proteins was found to alter gene expression in the diabetic heart and to be associated with DCM (2, 23, 35, 36). Abnormally elevated O-GlcNAc modification of endothelial nitric oxide synthase (eNOS) at Ser1177 deactivates eNOS and inhibits NO production (37), which impairs vasodilation, ECM remodeling and angiogenesis (**Figure 2C**). Importantly, several transcription factors, such as NF-κB (37, 38) and Sp1 (**Figure 2C**) (39), can be directly/indirectly activated by hyperglycemia-induced O-GlcNAcylation leading to the up-regulation of pro-inflammatory factors, including TGF-β, TNF-α and PAI-1, and down-regulation of SERCA2a leading to abnormal intracellular Ca^2+^ transient (38–40). Hyperglycemia clears anti-inflammatory and atheroprotective protein A20 *via* O-GlcNAcylation-dependent ubiquitination and proteasomal degradation (**Figure 2C**), which may be key to the cardiovascular system (37, 41).

### 3.2 Lipid metabolism disorder and DCM

**Figure 3:**
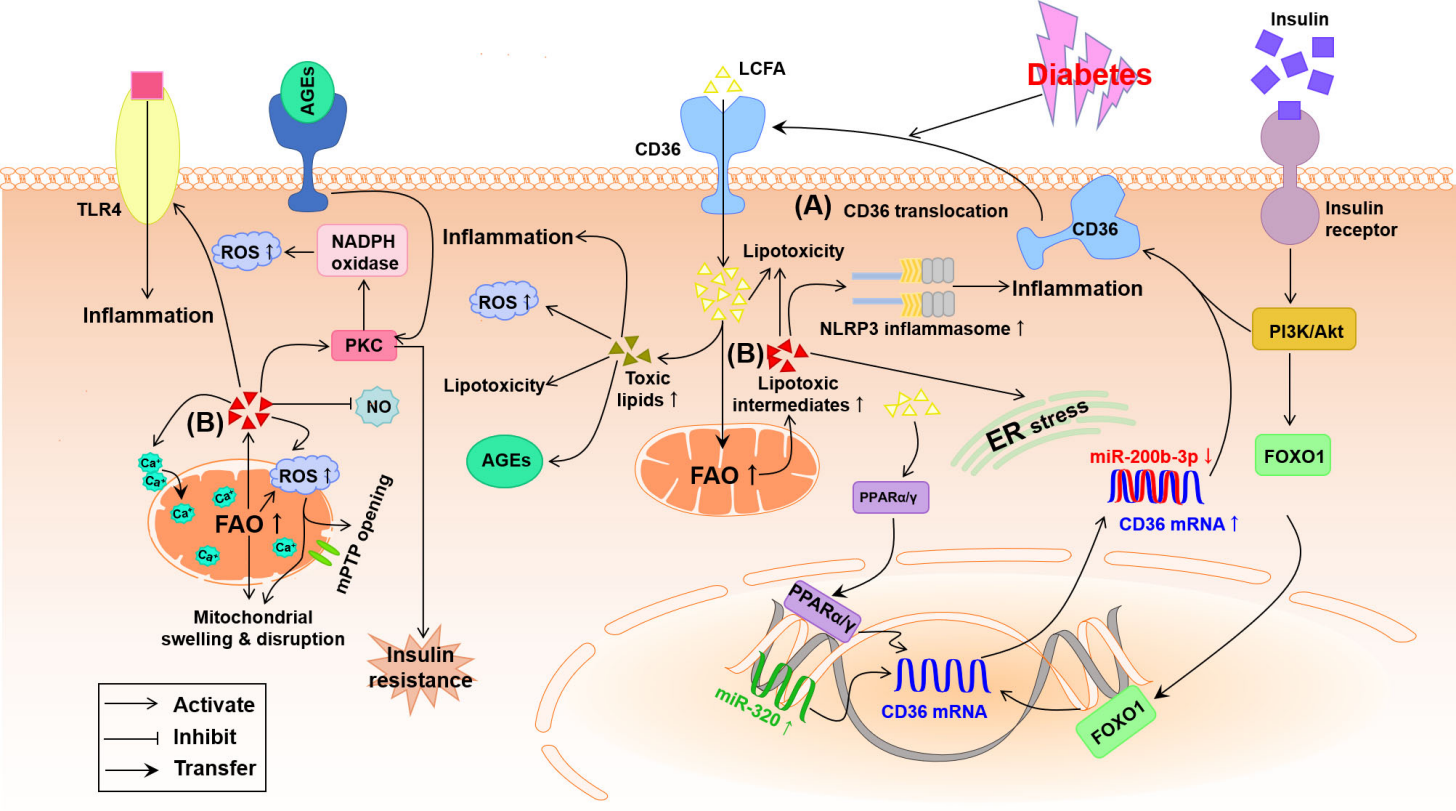
Potential pathways by which lipid metabolism disorder damages the diabetic heart. **(A)** Under diabetes conditions, the expression of cardiac PPARα/γ and FOXO1 is increased meanwhile miR-200b-3p is decreased and miR-320 is increased, which helps to up-regulation of CD36 causing the increase in LCFA uptake. **(B)** Abnormal FAO and accumulation of lipotoxic intermediates in the diabetic cardiomyocyte trigger oxidative stress, inflammation and ER stress. Furthermore, accumulation of lipotoxic intermediates in the diabetic cardiomyocyte increases mitochondrial uncoupling and impairment of mitochondria Ca^2+^ handing, leading to mitochondrial swelling and disruption through the opening of mitochondrial permeability transition pore (mPTP). AGEs, advanced glycation end-products; Akt, protein kinase B; CD36, cluster of differentiation 36; ER, endoplasmic reticulum; FAO, fatty acid oxidation; FOXO1, forkhead box protein O1; LCFA, long-chain fatty acid; NLRP3, nucleotide-binding domain, leucine-rich-containing family, pyrin domain containing 3; NO, nitric oxide; PI3K, phosphoinositide 3-kinase; PKC, protein kinase C; PPARα/γ, peroxisome proliferator-activated receptor α/γ; ROS, reactive oxygen species; TLR4, toll-like receptor 4.

Early in diabetes, abnormal glycometabolism grants the increase in fatty acid β-oxidation to compensate for a shortage of energy in the diabetic heart, which reduces mitochondrial oxidative capacity, and cardiac efficiency characterized by low ratio of myocardial ATP production/oxygen consumption and high mitochondria-derived superoxide anion (O_2_^-^) (42). As shown in **Figure 3**, the high fatty acid β-oxidation is accompanied with violent intracellular accumulation of toxic lipid metabolites, which precipitates DCM through multiple mechanisms including excessive generation of ROS, endoplasmic reticulum (ER) stress, and mitochondrial remodeling (43). Numerous studies using transgenic animal models have shown that up-regulation of myocardial fatty acid transporters such as CD36 and fatty acid transport protein 1 contribute to high fatty acid intake and lipotoxicity in the cardiomyocyte, which finally exacerbates the development of DCM (13, 44, 45).

#### 3.2.1 Up-regulation of CD36

The LCFA transporter CD36, also named as scavenger receptor B2, has been evidenced to take oxidized low-density lipoproteins (oxLDLs) and phospholipids into myocytes and lipocytes of rodents and humans, and up to 70% of total fatty acids into cardiomyocytes (46, 47). The up-regulation of CD36 has been evidenced on the sarcolemma of cardiomyocytes in diabetic mice and patients with DCM, which is owing to both high membrane translocation and high expression of CD36 triggered by hyperinsulinemia, hyperglycemia and hyperlipidemia during the development of diabetes (5, 48). As suggested in **Figure 3**, high insulin at the beginning of diabetes up-regulates CD36 mRNA expression by activating the transcription factor forkhead box protein O1 (FOXO1) and strongly transports CD36 to sarcolemma by activating the PI3K-Akt pathway while chronic high glucose and high triglyceride (TG) in the advanced stage of diabetes further facilitate CD36 expression and sarcolemma distribution (48, 49). The recent study indicates that up-regulated nuclear micro-RNA (miR)-320 and down-regulated miR-200b-3p induced by diabetes directly promote CD36 transcription and translation, respectively, thus further promoting the sarcolemma distribution of CD36 (**Figure 3**) (48). At the same time, excessive intracellular fatty acid accumulation and β-oxidation and high oxLDL uptake due to the increased sarcolemma distribution of CD36 produce a great deal of ROS, which can advance inflammation and insulin resistance to worsen DCM (4, 49–51). The cardiac impairment induced by the high level of CD36 has been further confirmed by the fact that absence of CD36 inhibited the accumulation of cardiac lipotoxicity whereas improved the cellular utilization of glucose ultimately rescuing DCM (52, 53). Therefore, the increased CD36 during DCM in turn aggravates the heart injury. In addition, the transcription factor peroxisome proliferator-activated receptor (PPAR), a class of ligand activated nuclear receptor including PPARα, PPARβ/δ and PPARγ, is activated by CD36-induced high intracellular LCFA, which up-regulates the transcription and sarcolemma distribution of CD36 to irritate fatty acids uptake and lipotoxicity in the diabetic myocardium through a positive feed-back (**Figure 3A**) (13, 54).

#### 3.2.2 Lipid accumulation-mediated myocardial injury

In the diabetic heart, insulin-dependent glucose intake is impaired while cardiac fatty acid influx and accumulation of lipids and lipotoxic intermediates are increased, resulting in cardiac lipotoxicity to play a causal role in the development of DCM (10, 13). Aberrant accumulation of lipids in diabetes usually leads to cardiovascular complications including DCM and heart failure (47). Excessive accumulation of lipids can facilitate numerous pathological processes linked to the development of DCM including mitochondrial dysfunction, ER stress, inflammation, and apoptosis (47, 55). The increase in myocardial fats especially TG (the other forms of LCFA) may exert deleterious effect on the left ventricular mass and diastolic function (56), which has been demonstrated in both ob/ob and db/db mice (57, 58). Furthermore, a constant myocardial influx of LCFA far exceeds cellular metabolic utilization, and the normal process of fatty acid β-oxidation is collapsed to generate plenty of mitochondrial derived ROS, which destroys cellular structure and function and promotes the process of DCM.

#### 3.2.3 Lipotoxic intermediates-mediated myocardial injury

Intramyocardial accumulation of lipotoxic intermediates, such as ceramide, diacylglycerol (DAG) and fatty acyl-CoAs, caused by lipid metabolism disorder creates a lipotoxic microenvironment in the heart, that likely renders much of the cardiac injuries in diabetes (47). As indicated in **Figure 3B**, these lipotoxic intermediates have been identified to activate some serine/threonine kinases including PKC and TLR4-mediated innate immunity, causing plenty of proinflammatory cytokines, oxidative stress, apoptosis and hypertrophy in the diabetic heart (47, 59, 60). Briefly, ceramide, the precursor of sphingolipids, stimulates the assembly of the nucleotide-binding domain, leucine-rich-containing family, pyrin domain containing 3 (NLRP3) inflammasome, ER stress, insulin resistance, and myocardial death (**Figure 3B**) (60, 61). DAG can activate PKC to trigger insulin resistance, the release of ROS and dilated lipotoxic cardiomyopathy. In addition, both DAG and ceramides probably inhibit the production of NO, which is responsible for cardiovascular endothelial dysfunction in diabetes (**Figure 3B**) (49). The increase of LCFA uptake exceeds the limited oxidation capacity in the diabetic heart, which causes the overproduction of fatty acyl-CoAs that associated with cardiac lipotoxicity (62). The high fatty acid oxidation (FAO) and excess fatty acyl-CoAs may activate PKCθ leading to insulin resistance and impaired glucose metabolism ultimately metabolic disorder leading to DCM (**Figure 3B**) (63, 64).

### 3.3 Common downstream pathway and miRNA interference

#### 3.3.1 PKC

PKC, a family of serine-threonine kinases composed of at least 12 isoforms, can phosphorylate target proteins and is essential for cell proliferation and differentiation in a tissue- and isoform-dependent manner (18, 65). Long-standing hyperglycemia-induced activation of PKC, through predominately a *de novo* synthesis of DAG, has been confirmed to thicken basement membrane, reduce blood flow and deposit ECM in the heart from both diabetic rodents and patients, which renders the development of DCM from cardiac inflammation, hypertrophy, fibrosis and diastolic dysfunction to heart failure (62, 66–69). Besides DAG, hyperglycemia may also activate PKC indirectly through increased flux of sorbitol pathway, increased flux of hexosamine pathway (**Figure 2**), and activated renin-angiotensin-aldosterone system, all of which result in the accumulation of pro-inflammatory cytokines and ROS in DCM (6, 34). PKC-induced overproduction of ROS mainly depends on the activation of NADPH oxidase (**Figures 2**, **3**), which has been verified by the fact that treatment with the inhibitor of PKC reduced NADPH oxidase-produced ROS and rescued the heart from DCM (70–72). In addition, PKC can up-regulate eNOS to produce peroxynitrite (ONOO^-^), a potent ROS, under the reaction of NO with O_2_^-^, which results in a decreased bioavailability of NO and an increased oxidative stress in the diabetic vasculature (**Figure 2**) (73). Furthermore, PKC can activate NF-κB *via* IκB kinase (IKK)-dependent inactivation of inhibitor of NF-κB to up-regulate proinflammatory factors (**Figure 2**) (74, 75). The role of PKC in the development of DCM has been verified by several studies that high oxidative stress and inflammation triggered by activation of PKCθ and PKCβ_2_ is essential to the diabetic cardiac hypertrophy and fibrosis (76–78).

#### 3.3.2 AMPK

AMP-activated protein kinase (AMPK) is an evolutionarily conserved serine-threonine kinase which regulates cellular energy homeostasis and coordinates multiple pathological processes, such as diabetes, cancer, cardiac hypertrophy and other chronic diseases (79, 80). Substantial evidence suggests that activating AMPK may be key to the function and survival of the diabetic cardiovascular system by balancing the utilization of intracellular energy substrates (such as glycolysis, TG synthesis and FAO) (80, 81), suppressing NLPR3 inflammasome-related inflammation and NADPH oxidase-related oxidative stress, and modulating autophagy and ER stress (82–88). In view of this, AMPK, for example, pharmacologically activated by metformin (80, 81, 86), appears to be a potential target for treating DCM.

#### 3.3.3 miRNAs

MiRNAs are a group of short (22~25 nucleotides), single‐stranded and highly conserved RNAs and commonly act as post-transcriptional inhibitors of gene expression (89). A variety of miRNAs, including miR-1, miR-152, miR-187, miR-208a, miR-802, miR-126 and so on, have identified to modulate insulin production, energy metabolism (89, 90), and oxidative stress (89) resulting in DCM characterized by hypertrophy (89, 91), fibrosis (92) and heart failure (93–97). Recent studies have shown that miR-21 is abundantly expressed in the diabetic hearts (98–100), which accounts for the high oxidative stress and inflammation-related DCM probably through the insufficient activation of nuclear factor-E2 related factor 2 signaling pathway (100) and sprouty homolog 1/extracellular signal-regulated kinase/mammalian target of rapamycin (SPRY1/ERK/mTOR) pathway (99). The expression of miRNAs, such as miR-146a and miR-221, is disturbed to induce an enhancement of oxidative stress and inflammation response, which is acknowledged to promote the development of DCM. Several studies have showed that the significantly down-regulated miR-146a induced by longstanding hyperglycemia not only causes the overexpression of pro-inflammatory factors (IL-6, TNF-α and IL-1β) and NF-κB (101–103) but also generates excess ROS *via* NADPH oxidase 4 pathway, which impairs the diabetic cardiovascular system (101).

## 4 Inflammatory response and oxidative stress in DCM

**Figure 4:**
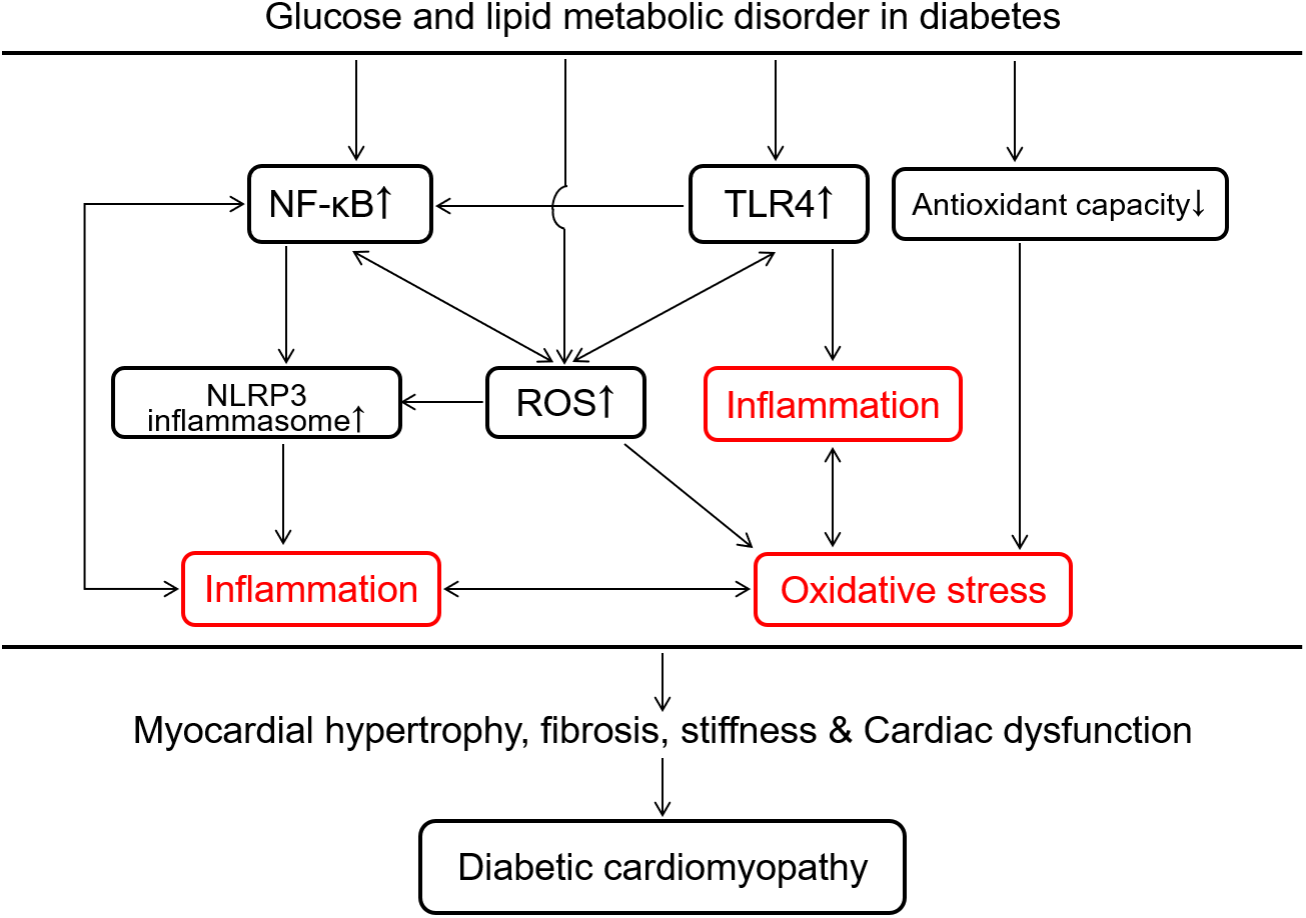
Overview of intracellular inflammation and oxidative stress interaction in response to glucose and lipid metabolic disorder in the development of diabetic cardiomyopathy. Glucose and lipid metabolic disorder in diabetes promotes cardiac inflammation *via* TLR4-mediated innate immunity, the surge of reactive oxygen species (ROS) and NF-κB-induced activation of NLRP3 inflammasome. The decrease in the antioxidant capacity and the increase in ROS production induced by the metabolic disorder both accelerate oxidative stress in the diabetic heart. Inflammation and oxidative stress promote each other to cause myocardial hypertrophy, fibrosis, stiffness and cardiac dysfunction, which is a potentially vicious cycle in the development of diabetic cardiomyopathy. NF-κB, nuclear factor κ-light-chain-enhancer of activated B cells; NLRP3, nucleotide-binding domain, leucine-rich-containing family, pyrin domain containing 3; TLR4, toll-like receptor 4.

Metabolic disturbance in the context of diabetes is associated with inflammation characterized by the excessive release of pro-inflammatory cytokines and activation of inflammatory cascade response (104). Pro-inflammatory cytokines, especially TNF-α, can lead to cardiac remodeling and dysfunction in the progression of diabetes. TNF-α inhibits tyrosine phosphorylation of insulin receptor substrate-1 aggravating insulin resistance and increases ventricular hypertrophy and vascular permeability leading to heart failure in diabetes (105). Inflammation and oxidative stress are closely interlinked (**Figure 4**). Both inflammatory response and overproduction of ROS/reactive nitrogen species (RNS) induced by metabolic disturbance in diabetes trigger NF-κB transcription and translocation to further arise pro-inflammatory cytokines and ROS/RNS, forming a vicious cycle of diabetic complications (106). Hyperglycemia and the increase of FAO produce excessive ROS/RNS to modify and blunt endogenous antioxidant defense systems, leading to deadly cell abnormalities such as mitochondrial membrane potential disruption (107), DNA double-strand break (27), and ER stress in diabetes (108). Eliminating pro-inflammatory factors and ROS/RNS is involved in the protection of correcting metabolic disturbance against DCM, which has been demonstrated by the curative effect of statins and active compounds in medicinal plants in diabetic heart diseases (109, 110).

### 4.1 Inflammation in DCM

A chronic inflammatory response clearly persists in diabetes through deadly activating the innate immune system to damage the heart (20, 111). Some early-responding pro-inflammatory immune cells, like M1-polarized macrophages, lymphocytes and neutrophils, secrete IL-6, IL-18, TNF-α, IL-1β to attack vascular smooth muscle cells, fibroblasts and cardiomyocytes impairing cellular energy metabolism and cause cardiovascular dysfunction (112, 113). TLR4 can be activated by the excess free fatty acid and pathogen-associated molecular patterns (PAMPs) to induce the synthesis of pro-inflammatory cytokines, meanwhile the activation of NF-κB by TLR4 also amplify the inflammatory response and pathological alterations (**Figure 4**) (114). This up-regulation of TLR4-mediated inflammation has been found in the diabetic heart (115) and repression of TLR4 benefits to lower lipid accumulation and to ameliorate cardiac function in diabetes (13, 116). Additionally, NLRP3 inflammasome, assembled by NLRP3, caspase-1, and apoptosis-associated speck-like protein containing a caspase recruitment domain (ASC), has been considered as another inflammatory participant in the ventricular remodeling and dysfunction of cardiac disease such as acute myocardial infarction, DCM and heart failure (117–119). The deleterious effect of activating NLRP3 inflammasome in DCM includes inflammation, fibrosis, pyroptosis and apoptosis (119–122). In addition to DAMPs/PAMPs (119, 123, 124), the increased fatty acids (122, 125), lipid intermediates (122), glucose (119), ROS (37, 119) are also the positive regulator to boost NLRP3 inflammasome in DCM. Once exposed to diabetic or other nociceptive stimuli, the auto-oligomerization of NLRP3 inflammasome is rapidly initiated by connecting NLRP3 proteins with ASC through both PYD domains (119). The NLRP3-ASC complex recruits pro-caspase-1 to form NLRP3 inflammasome *via* CARD domain interaction (122). Subsequently, pro-caspase-1 is cleaved into activated caspase-1, which promotes the secretion of IL-1β and IL-18 leading to a novel cell death named pyroptosis (123, 126). NLRP3 inflammasome plays a vital role in the progression of inevitable cardiac fibrosis and collagen deposition *via* suppressing MAPK signaling pathway and the production of cAMP and in myocardial fibroblasts (127–129). Given such destructive effect, NLRP3 inflammasome is probably a new target for the treatment of DCM.

### 4.2 Oxidative stress in DCM

ROS are primarily produced by mitochondrial respiratory chain as an electron leakage due to the impairing electron transport in hyperglycemia (130). NADPH oxidase is another prominent source of ROS in diabetes (27, 131). The decline of endogenous antioxidant capacity by inactivating antioxidants (such as GSH and vitamin E) and/or down-regulating antioxidant enzymes (such as superoxide dismutase, peroxidase and catalase) is also involved in the oxidative stress to develop DCM (**Figure 4**) (2, 27, 71, 130). Excess ROS can break DNA double-strand and oxidize proteins, by which glyceraldehyde 3-phosphate dehydrogenase, a key glycolytic enzyme in the glycolysis process, is down-regulated leading to the inhibition of glycolysis and accumulation of glycolytic intermediates (37). High glucose powerfully hampers the pentose phosphate pathway *via* inhibiting its rate-limiting enzyme glucose-6-phosphate dehydrogenase, to decrease the production of NADPH and GSH resulting in a lower antioxidant defense with a higher ROS production in endothelial cells and cardiomyocytes (**Figure 1**) (132, 133). The excessive ROS in diabetes can modify the structure of lipids named lipid peroxidation such as formation of malondialdehyde, 4-hydroxynonenam and oxLDL. These oxidized lipids commonly enhance pro-inflammatory response and participate diabetic cardiovascular complications including atherosclerosis and DCM (50, 51). Hence, oxidative stress represents a major force in the development of diabetic inflammation and probably an important target in the treatment of DCM accompanied by disproportionate inflammation (**Figure 4**).

## 5 Conclusion

A variety of studies have confirmed that glucose and lipid metabolic disturbance plays a central role in the development of DCM through vicious oxidative stress and inflammatory response pathways. The polyol pathway, hexosamine biosynthesis, PCK activation, sarcolemmal translocation of CD36, up-regulation of AGE/RAGE system, and disequilibrium of micro-RNA are involved in the metabolic disturbance, all of which contribute to the enhancement of diabetic oxidative stress and inflammation finally resulting in DCM. Although several dependable first-line drugs targeting glucose/lipid metabolism such as metformin, thiazolidinediones and sodium-glucose transporter-2 inhibitors alleviate hyperglycemia, the exact effect of these agents on DCM still needs to be further explored in the diabetic animal model and patient.

## Author contributions

M-YC wrote the manuscript. M-YC, X-FM, Y-PH, J-LY, CX, and L-BQ designed the figures and edited the manuscript. CX and L-BQ supervised the writing. All authors contributed to the article and approved the submitted version.

## Funding

This work was supported by National Natural Science Foundation of China (81772035), Natural Science Foundation of Zhejiang Province (LQ20H150001), and Program of Cultivating Zhejiang Provincial High-level Personnel in Health (Innovative Talent in 2021).

## Conflict of interest

The authors declare that the research was conducted in the absence of any commercial or financial relationships that could be construed as a potential conflict of interest.

## Publisher’s note

All claims expressed in this article are solely those of the authors and do not necessarily represent those of their affiliated organizations, or those of the publisher, the editors and the reviewers. Any product that may be evaluated in this article, or claim that may be made by its manufacturer, is not guaranteed or endorsed by the publisher.

## Abbreviations

AR, Aldose reductase; AGEs, Advanced glycation end-products; AMPK, AMP-activated protein kinase; ASC, Apoptosis-associated speck-like protein containing a caspase recruitment domain; CD36, Cluster of differentiation 36; DAG, Diacylglycerol; DCM, Diabetic cardiomyopathy; ECM, Extracellular matrix; eNOS, Endothelial nitric oxide synthase; ER, Endoplasmic reticulum; FAO, Fatty acid oxidation; F-6-P, Fructose 6-phosphate; FOXO1, Forkhead box protein O1; Glucosamine-6-P, Glucosamine-6-phosphate; GFAT, Glutamine-fructose-6-phosphate amidotransferase; GLUTs, Glucose transporters; GSH, Reduced glutathione; GSSH, Oxidized glutathione; IKK, IκB kinase; LCFA, Long-chain fatty acids; mPTP, Mitochondrial permeability transition pore; NAD^+^, Oxidized form of nicotinamide adenine dinucleotide; NADPH, Reduced form of nicotinamide adenine dinucleotide phosphate; NF-κB, Nuclear factor κ-light-chain-enhancer of activated B cells; NLRP3, Nucleotide-binding domain, leucine-rich-containing family, pyrin domain containing 3; O^2-^, Superoxide anion; O-GlcNAcylation, O-linked N-acetylglucosamine glycosylation; oxLDL, Oxidized low-density lipoprotein; PAMPs, Pathogen-associated molecular patterns; PKC, Protein kinase C; PPAR, Peroxisome proliferator-activated receptor; RAGE, Receptor for AGE; ROS, Reactive oxygen species; T1DM, Type 1 diabetes mellitus; TLR4, Toll-like receptor 4; UDP-GlcNAc, UDP-N-acetylglucosamine.
